# Angiotensin II receptor type 1 blockade regulates Klotho expression to induce TSC2-deficient cell death

**DOI:** 10.1016/j.jbc.2022.102580

**Published:** 2022-10-08

**Authors:** Shikshya Shrestha, Elio Adib, Jewel Imani, Dean J. Aguiar, Anthony M. Lamattina, Dereje D. Tassew, Elizabeth P. Henske, Mark A. Perrella, Carmen Priolo, Souheil El-Chemaly

**Affiliations:** 1 Division of Pulmonary and Critical Care Medicine, Brigham and Women’s Hospital, Harvard Medical School, Boston, Massachusetts, USA; 2 TSC Alliance, Silver Spring, Maryland, USA

**Keywords:** angiotensin II, Agtr1, klotho, LAM, losartan, tuberin, receptor, cell death, signaling, ACE, angiotensin-I converting enzyme, AGTR, angiotensin II receptor, AML, angiomyolipomas, cPARP, cleaved PARP, DMSO, dimethyl sulfoxide, EV, empty vector, KD, knockdown, LAM, lymphangioleiomyomatosis, LDH, lactate hydrogenase, MEF, mouse embryonic fibroblasts, mTORC1, mammalian/mechanistic target of Rapamycin complex 1, NT-shRNA, nontarget shRNA, qPCR, quantitative PCR, RAS, renin-angiotensin system, sKlotho, soluble Klotho, TSC, tuberous sclerosis complex

## Abstract

Lymphangioleiomyomatosis (LAM) is a multisystem disease occurring in women of child-bearing age manifested by uncontrolled proliferation of smooth muscle–like “LAM” cells in the lungs. LAM cells bear loss-of-function mutations in tuberous sclerosis complex (TSC) genes *TSC1* and/or *TSC2*, causing hyperactivation of the proliferation promoting mammalian/mechanistic target of Rapamycin complex 1 pathway. Additionally, LAM-specific active renin-angiotensin system (RAS) has been identified in LAM nodules, suggesting this system potentially contributes to neoplastic properties of LAM cells; however, the role of this renin-angiotensin signaling is unclear. Here, we report that TSC2-deficient cells are sensitive to the blockade of angiotensin II receptor type 1 (Agtr1). We show that treatment of these cells with the AGTR1 inhibitor losartan or silencing of the *Agtr1* gene leads to increased cell death *in vitro* and attenuates tumor progression *in vivo*. Notably, we found the effect of Agtr1 blockade is specific to TSC2-deficient cells. Mechanistically, we demonstrate that cell death induced by Agtr1 inhibition is mediated by an increased expression of Klotho. In TSC2-deficient cells, we showed overexpression of Klotho or treatment with recombinant (soluble) Klotho mirrored the cytocidal effect of angiotensin blockade. Furthermore, Klotho treatment decreased the phosphorylation of AKT, potentially leading to this cytocidal effect. Conversely, silencing of Klotho rescued TSC2-deficient cells from cell death induced by Agtr1 inhibition. Therefore, we conclude that Agtr1 and Klotho are important for TSC2-deficient cell survival. These findings further illuminate the role of the RAS in LAM and the potential of targeting Agtr1 inhibition in TSC2-deficient cells.

Tuberous sclerosis complex (TSC) is an autosomal dominant syndrome characterized by hamartoma-like tumor growths in various organs, cerebral calcifications, seizures, and mental retardation, and the development of cystic lung disease called lymphangioleiomyomatosis (LAM). TSC is caused when one of the TSC genes *TSC1* or *TSC2* is mutated, more often *TSC2* than *TSC1* (1, 2), resulting in increased activation of the mammalian/mechanistic target of Rapamycin complex 1 (mTORC1) (3, 4, 5, 6). LAM is a multisystem disorder primarily affecting women of child-bearing age, characterized by cystic lung destruction, axial lymphatic abnormalities, and abdominal angiomyolipomas (AML) (7, 8, 9, 10, 11, 12, 13). LAM occurs sporadically in patients with no evidence of germline genetic abnormality and by the age of 40 in about 80% of women with TSC (14, 15, 16). Inhibitors of the mTORC1 pathway have been shown to have therapeutic benefits in LAM and other TSC manifestations; however, there is a need for continuous therapy for persistent benefit, since mTORC1 inhibitors have cytostatic, and not cytotoxic, effects on TSC2-deficient cells (17, 18). Identifying novel therapies that alone or in combination with mTORC1 inhibitors can induce TSC2-deficient cell death is of critical importance. We recently demonstrated that targeting E26 transformation–specific (ETS) variant transcription factor 2, an ETS family transcription factor specifically in TSC2-deficient cell promotes cytocidal response *via* regulation of poly(ADP-ribose) polymerase (PARP)-1 binding protein and therefore can have therapeutic potential in LAM or other TSC manifestations (19).

Multiple TSC-related manifestations have also been linked to the renin-angiotensin system (RAS). For instance, LAM lung nodules have been shown to possess a functional RAS, including the presence of renin, angiotensin I converting enzyme (ACE), angiotensinogen, angiotensin II, and angiotensin II receptors (20). A retrospective review of LAM patients receiving ACE inhibitors showed slower decline in lung function (21). Furthermore, patients with TSC2-polycystic kidney disease 1 deletion syndrome and hypertension treated with inhibition of the RAS pathway *via* angiotensin receptor blockade had decreased renal AML development compared to those who did not receive this therapy (22). However, the precise mechanisms underpinning these effects of angiotensin II receptor (AGTR) blockade have not been elucidated. Here, we seek to exploit the RAS present in LAM or TSC to overcome the limitations of rapalogs.

*Klotho*, an aging-suppressor gene, encodes for α-Klotho protein (Klotho), which associates with fibroblast growth factor receptor family protein to regulate metabolic processes (23, 24, 25). Klotho is a transmembrane protein with a short cytoplasmic domain and two large homologous extracellular domains, KL1 and KL2. The proteolytic cleavage of extracellular domains by beta-secretase generates the soluble form of Klotho that is detected in circulation in various bodily fluids (26, 27, 28, 29, 30, 31). Soluble Klotho (sKlotho) has been associated with regulation of transporters, oxidative stress, and signaling factors, as well as inhibition of insulin-like growth factor-1 receptor (32). Klotho’s function as a tumor suppressor has also been demonstrated in various malignancies, including colorectal, pancreatic, gastric, renal, breast, and ovarian cancers (23, 24, 33, 34, 35, 36, 37, 38). Furthermore, multiple lines of evidence show that Klotho is downstream of both mTORC1 and angiotensin signaling (39, 40, 41, 42, 43, 44). However, the role of Klotho in TSC2-related manifestations remains to be examined.

Herein, we explored the roles of AGTR1 and Klotho in TSC2-deficient cells. We show that blocking AGTR1 in TSC2-deficient cells results in cell death *in vitro* and inhibition of tumor growth *in vivo*. Crucially, we also show that treatment with losartan, an AGTR1-blocker, as well as shRNA-mediated *Agtr1* knockdown (KD) leads to induction of Klotho expression. Further, genetic manipulation of Klotho (silencing or overexpressing) leads to protection against or induction of cell death, respectively. Additionally, treatment with sKlotho increased TSC2-deficient cell death by reducing AKT phosphorylation. Based on our data, Klotho-dependent cytotoxic effect of AGTR1 blockade may highlight a potential therapeutic target for the treatment of LAM and other manifestations of TSC.

## Results

### AGTR1 expression is TSC2 loss–independent

Tuberin deficiency results in mTORC1 hyperactivation, leading to uncontrolled cell growth (45). As we hypothesize that AGTR1 is involved in LAM pathogenesis, we first sought to determine whether AGTR1 expression is regulated upon *Tsc2* loss. We performed quantitative PCR (qPCR) and Western blot using TSC2-deficient ELT3V and TSC2-addback ELT3T cells derived from Eker rat uterine leiomyoma (46). Both cell types expressed *Agtr1* mRNA and AGTR1 protein at similar levels, suggesting that AGTR1 expression is *Tsc2* loss–independent (Fig. S1, *A*–*D*).

### AGTR1 inhibition induces TSC2-deficient cell death *in vitro*

**Figure 1:**
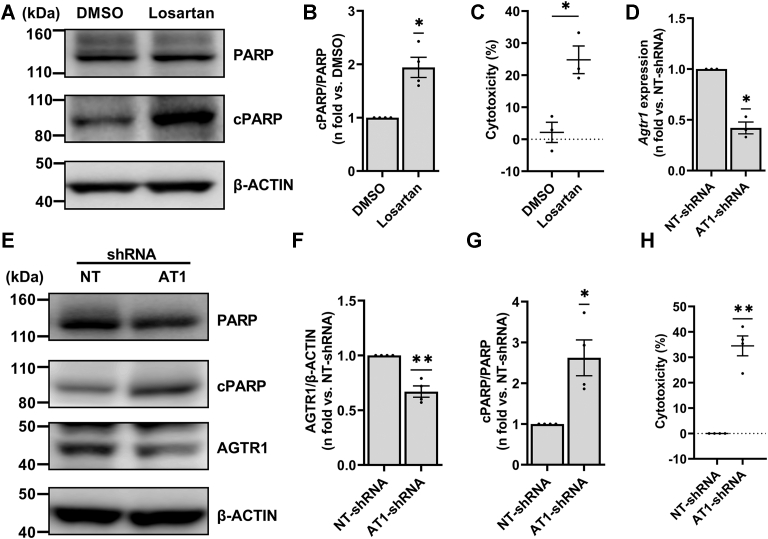
**Inhibition of AGTR1 induces TSC2-deficient cell death *in vitro***. *A*, TSC2-deficient ELT3V cells were starved overnight and treated with DMSO (vehicle control) or losartan (100 nM) for 24 h in 0.5% serum supplemented media. Equal amounts of protein from whole-cell lysates of treated cells were analyzed by Western blot. Representative blots for PARP, cleaved PARP (cPARP), and β-ACTIN (loading control) are shown. *B*, histogram for cPARP/PARP is presented as the fold change relative to DMSO-treated ELT3V cells. *C*, scatter plot for LDH release by ELT3V cells treated with DMSO or losartan is presented as percent cytotoxicity. No drug (or water control) samples were used as “low control” for LDH measurement. *D*, Sh-RNA–mediated *Agtr1* knockdown (AT1-shRNA) in ELT3V cells. *D*, quantitative PCR analysis of shRNA-mediated knockdown of *Agtr1.* Histogram for *Agtr1* mRNA expression in ELT3V cells targeted with control (NT-shRNA) and AT1-shRNA is presented. *B2m* was used as a housekeeping gene. *E*, representative blots for AGTR1, cPARP, PARP, and β-ACTIN (loading control). Equal amounts of protein from whole-cell lysates of NT-shRNA and AT1-shRNA cells were analyzed by Western blot. Histograms for (*F*) AGTR1/β-ACTIN and (*G*) cPARP/PARP are expressed as the fold change relative to NT-shRNA cells. *H*, scatter plot for LDH release by NT-shRNA and AT1-shRNA presented as percent cytotoxicity. NT-shRNA samples were used as “low control” for LDH measurement. All graphs represent mean ± SEM of at least three independent experiments. Each biological replicate value is presented as a full circle. Statistical significance of ∗*p* < 0.05 or ∗∗*p* < 0.01 was determined by (*B*, *D*, and *F–H*) one-sample *t* test or (*C*) two-tailed *t* test. AGTR, angiotensin II receptor; cPARP, cleaved PARP; DMSO, dimethyl sulfoxide; LDH, lactate hydrogenase; NT-shRNA, nontarget shRNA; PARP, poly(ADP-ribose) polymerase; TSC, tuberous sclerosis complex.

Next, to determine the effects of AGTR1 blockade on TSC2-deficient cells, we treated ELT3V cells with losartan and examined the effects on cell survival. Treatment with losartan resulted in a decrease in P38 MAPK phosphorylation, a known downstream effector of AGTR1 signaling (Fig. S2, *A* and *B*) (47). Western blot analysis demonstrated an increase in cleaved PARP (cPARP) levels compared to dimethyl sulfoxide (DMSO) control, suggesting increased cell death (Fig. 1, *A* and *B*). We further evaluated the levels of Caspase7, an upstream regulator of PARP cleavage (48, 49) and found that treatment with losartan increased cleaved Caspase7 levels (Fig. S2, *C* and *D*). Additionally, treatment with losartan induced a significant increase in lactate dehydrogenase (LDH) release, suggesting disruption of the cell membrane and increased cytotoxicity (Fig. 1*C*). The deep blue cell viability assay also confirmed that losartan treatment led to reduced TSC2-deficient cell viability (Fig. S2*E*). To demonstrate that these effects are specific to TSC2-deficient cells, we treated TSC2-addback ELT3T cells with losartan and showed that in contrast to the effects of AGTR1 blockade in ELT3V cells, ELT3T cells treated with losartan did not have an increase in cell death as demonstrated by the lack of increase in cPARP level (Fig. S3, *A* and *C*) as well as no difference in LDH release compared to DMSO treatment (Fig. S3*D*).

Since losartan can also affect transforming growth factor-β signaling in a nonreceptor-dependent manner (50, 51, 52, 53) and to ascertain that the observed effects of losartan on TSC2-deficient cell death are driven by the AGTR1, we silenced *Agtr1* using shRNA in ELT3V cells. *Agtr1* KD cells, denoted by AT1-shRNA, showed significantly reduced *Agtr1* mRNA and AGTR1 protein expressions compared to nontarget control cells (NT-shRNA) (Fig. 1, *D*–*F*). Like ELT3V cells treated with losartan, AT1-shRNA cells demonstrated increased level of cPARP expression compared to NT-shRNA cells (Fig. 1, *E* and *G*), increased LDH release (Fig. 1*H*), and decreased cell viability (Fig. S2*F*). Together, these data strongly suggest that *Agtr1* gene silencing leads to increased TSC2-deficient cell death.

These data demonstrate that, contrary to TSC2-addback cells, TSC2-deficient cells require continuous AGTR1 signaling for cell survival.

### AGTR1 regulates Klotho expression in TSC2-deficient cells

**Figure 2:**
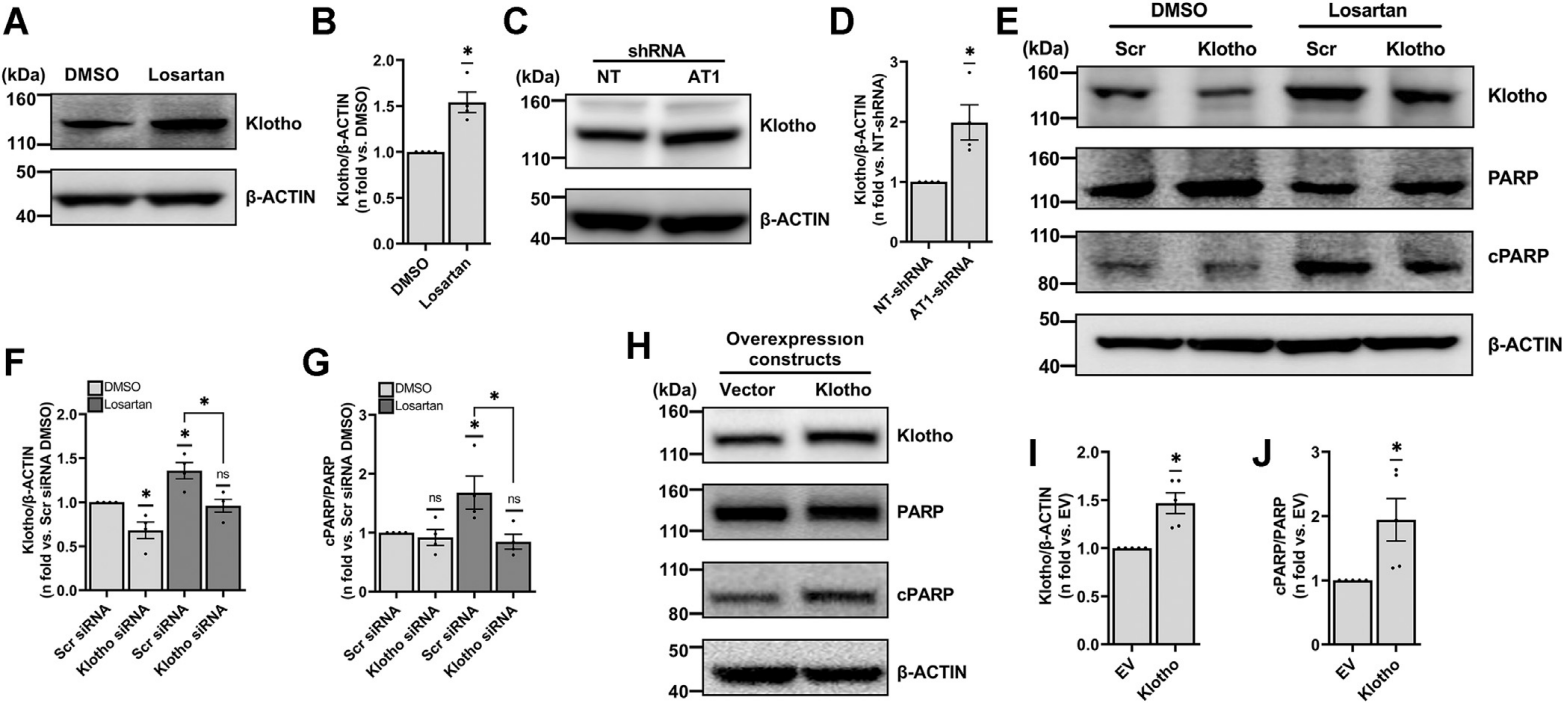
**AGTR1 blockade–mediated TSC2-deficient cell death is Klotho-dependent**. *A*, TSC2-deficient ELT3V cells were starved overnight and treated with DMSO or losartan (100 nM) for 24 h in 0.5% serum supplemented media. Equal amounts of protein isolated from whole-cell lysates of treated cells were analyzed by Western blot. Representative blots for Klotho and β-ACTIN (same membrane as Fig. 1*A*) are shown. *B*, histogram for Klotho/β-ACTIN is presented as the fold change relative to DMSO-treated ELT3V cells. *C*, equal amounts of lysates extracted from NT-shRNA and AT1-shRNA ELT3V cells were analyzed by Western blot. Representative blots for Klotho and β-ACTIN (same membrane as Fig. 1*E*) are shown. *D*, histogram for Klotho/β-ACTIN is presented as the fold change relative to NT-shRNA–treated ELT3V cells. *E*, ELT3V cells were transfected with Scr or Klotho siRNA, starved overnight, and treated with losartan (100 nM) for 24 h. Equal amounts of lysates were analyzed by Western blot. Representative blots for Klotho, cPARP, PARP, and β-ACTIN (*loading control*) are shown. Histograms for (*F*) Klotho/β-ACTIN and (*G*) cPARP/PARP are presented as fold change relative to Scr siRNA-transfected cells treated with DMSO. *H*, ELT3V cells were transfected with an empty vector or Klotho construct for 48 h. Equal amounts of lysates extracted were subjected to Western blot analysis. Representative blots for Klotho, cPARP, PARP, and β-ACTIN (*loading control*) are shown. Histogram for (*I*) Klotho/β-ACTIN and (*J*) cPARP/PARP are presented as fold change relative to vector transfected cells. All graphs represent mean ± SEM of at least three independent experiments. Each biological replicate value is presented as a full circle. Statistical significance of ∗*p* < 0.05 for each graph was determined by one-sample *t* test. AGTR, angiotensin II receptor; cPARP, cleaved PARP; DMSO, dimethyl sulfoxide; NT-shRNA, nontarget shRNA; PARP, poly(ADP-ribose) polymerase; TSC, tuberous sclerosis complex.

Angiotensin receptor blockade has been shown to induce Klotho expression in the kidney (44, 54). Like *Agtr1*, comparison between TSC2-deficient ELT3V and TSC2-addback ELT3T cells showed no significant differences in Klotho expression (Fig. S1, *B* and *D*). Next, to evaluate whether Klotho is downstream of AGTR1 in TSC2-deficient cells, we treated ELT3V and ELT3T cells with losartan and analyzed cell lysates for Klotho expression with Western blotting. Our data showed that AGTR1 inhibition with losartan resulted in increased Klotho levels only in TSC2-deficient cells (Fig. 2, *A* and *B*) but not in TSC2-addback cells (Fig. S3, *A* and *B*). Further, to confirm that these effects are AGTR1-dependent, we examined Klotho protein levels in *Agtr1* KD cells and found a corresponding increase in Klotho levels in AT1-shRNA cells compared to NT-shRNA cells (Fig. 2, *C* and *D*). To confirm the specificity of AT1-shRNA, a second construct (AT1-shRNA-2) was used to corroborate that *Agtr1* silencing leads to increased Klotho expression and cell death (Fig. S4, *A*–*E*). Taken together, our data show that Klotho is downstream of the AGTR1 receptor in TSC2-deficient cells.

### AGTR1-dependent cell death is driven by Klotho in TSC2-deficient cells

To elucidate Klotho’s involvement in *Agtr1* blockade–mediated cell death, we silenced *Klotho* using siRNA in ELT3V cells (Fig. 2, *E* and *F*), followed by treatment with losartan. We observed that cells targeted with Klotho siRNA had a marked decrease in cPARP compared to cells targeted with Scr siRNA (Fig. 2, *E* and *G*), suggesting that Klotho is necessary for AGTR1 blockade–dependent cell death in TSC2-deficient cells. These results were also confirmed with a second Klotho siRNA (Fig. S4, *F*–*H*).

Finally, to examine if increased Klotho levels are sufficient to induce TSC2-deficient cell death, ELT3V cells were transiently transfected with Klotho or empty vector (EV) construct for 48 h and subjected to Western blotting. Cells overexpressing Klotho demonstrated increased cell death as shown by the increase in cPARP levels compared to vector transfected cells (Fig. 2, *H*–*J*). Taken together, these data show that Klotho is necessary and sufficient to induce TSC2-deficient cell death and that AGTR1 signaling is necessary for mTORC1-hyperactive cell survival.

**Figure 3:**
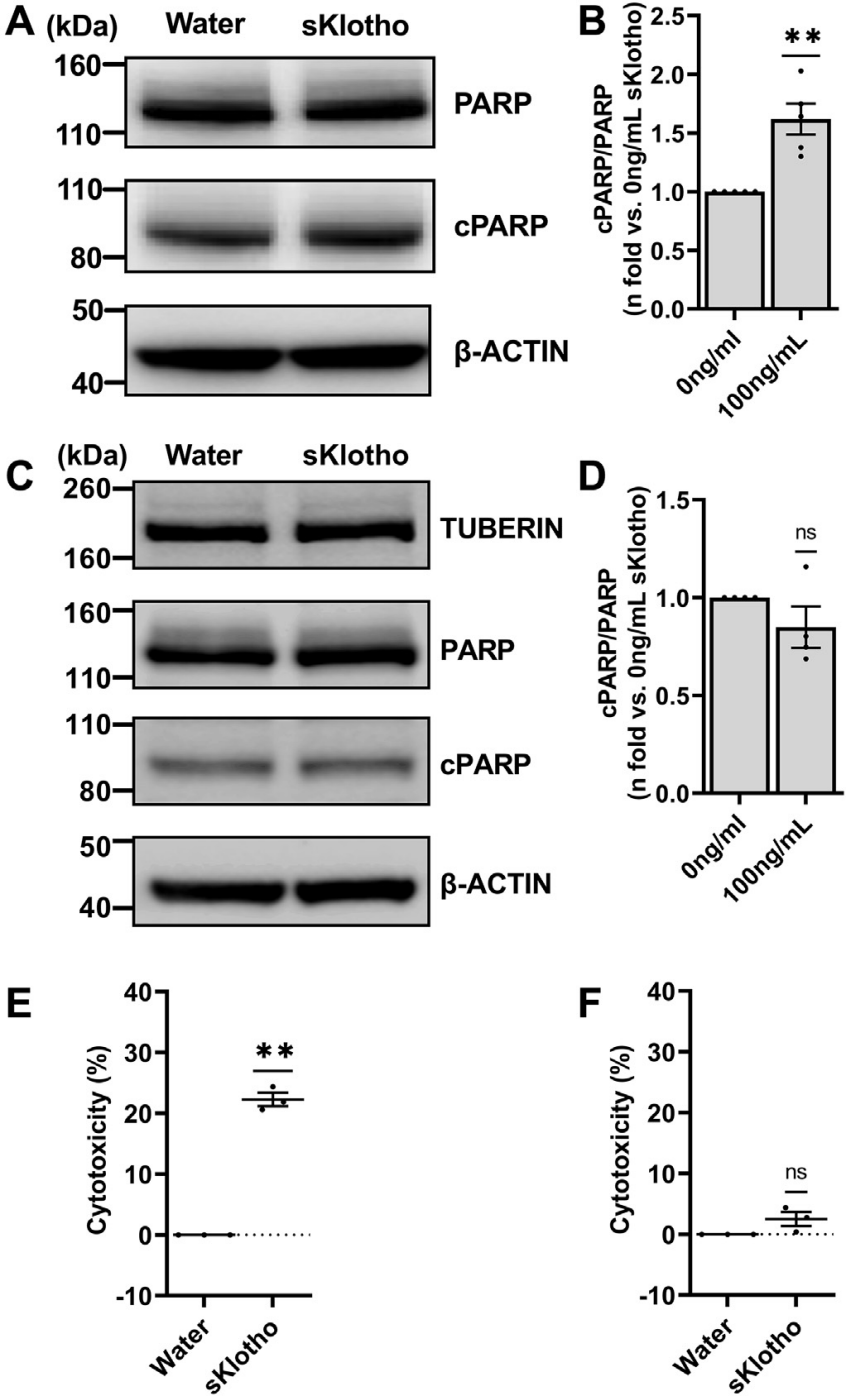
**Soluble Klotho treatment induces cell death only in TSC2-deficient cells but not TSC2-addback cells**. Representative blots for cPARP, PARP, TUBERIN, and β-ACTIN (loading control) in (*A*) TSC2-deficient ELT3V cells and (*C*) TSC2-addback ELT3T cells starved overnight and then treated with vehicle control (water, 0 ng/ml sKlotho) or sKlotho (100 ng/ml) for 24 h in serum starved conditions are shown. Equal amounts of lysates extracted from treated cells were subjected to Western blot analysis. Histograms for cPARP/PARP expression in (*B*) ELT3V and (*D*) ELT3T cells are presented as fold change relative to control (0 ng/ml sKlotho). Scatter plot of LDH release presented as percent cytotoxicity after exposure of (*E*) ELT3V and (*F*) ELT3T cells to water and sKlotho treatment. Water-treated samples are used as “low control” for percent cytotoxicity calculation. All graphs represent mean ± SEM of at least three independent experiments. Each biological replicate value is presented as a full circle. Statistical significance of ns *p* > 0.05 or ∗∗*p* < 0.01 were determined by one-sample *t* test. cPARP, cleaved PARP; LDH, lactate hydrogenase; PARP, poly(ADP-ribose) polymerase; sKlotho, soluble Klotho; TSC, tuberous sclerosis complex.

Next, we wanted to examine if sKlotho affects TSC2-deficient cell survival. First, we performed an LDH release assay with increasing sKlotho concentrations and determined that 100 ng/ml was the optimal dose (Fig. S2*G*), which is consistent with previous studies (55, 56). Next, we exposed ELT3V cells with 100 ng/ml sKlotho for 24 h. Treatment with sKlotho significantly increased cPARP level, suggesting increased cell death (Fig. 3, *A* and *B*). Similar to AGTR1 inhibition, treatment of TSC2-addback ELT3T cells with sKlotho did not result in cell death (Fig. 3, *C* and *D*). Treatment with sKlotho also induced a significant increase in LDH release in TSC2-deficient ELT3V cells but not in TSC2-addback ELT3T cells (Fig. 3, *E* and *F*). Finally, sKlotho-driven cell death was also confirmed by demonstrating the decreased cell viability in TSC2-deficient cells (Fig. S2*H*).

### Klotho-dependent cell death is mediated by AKT

**Figure 4:**
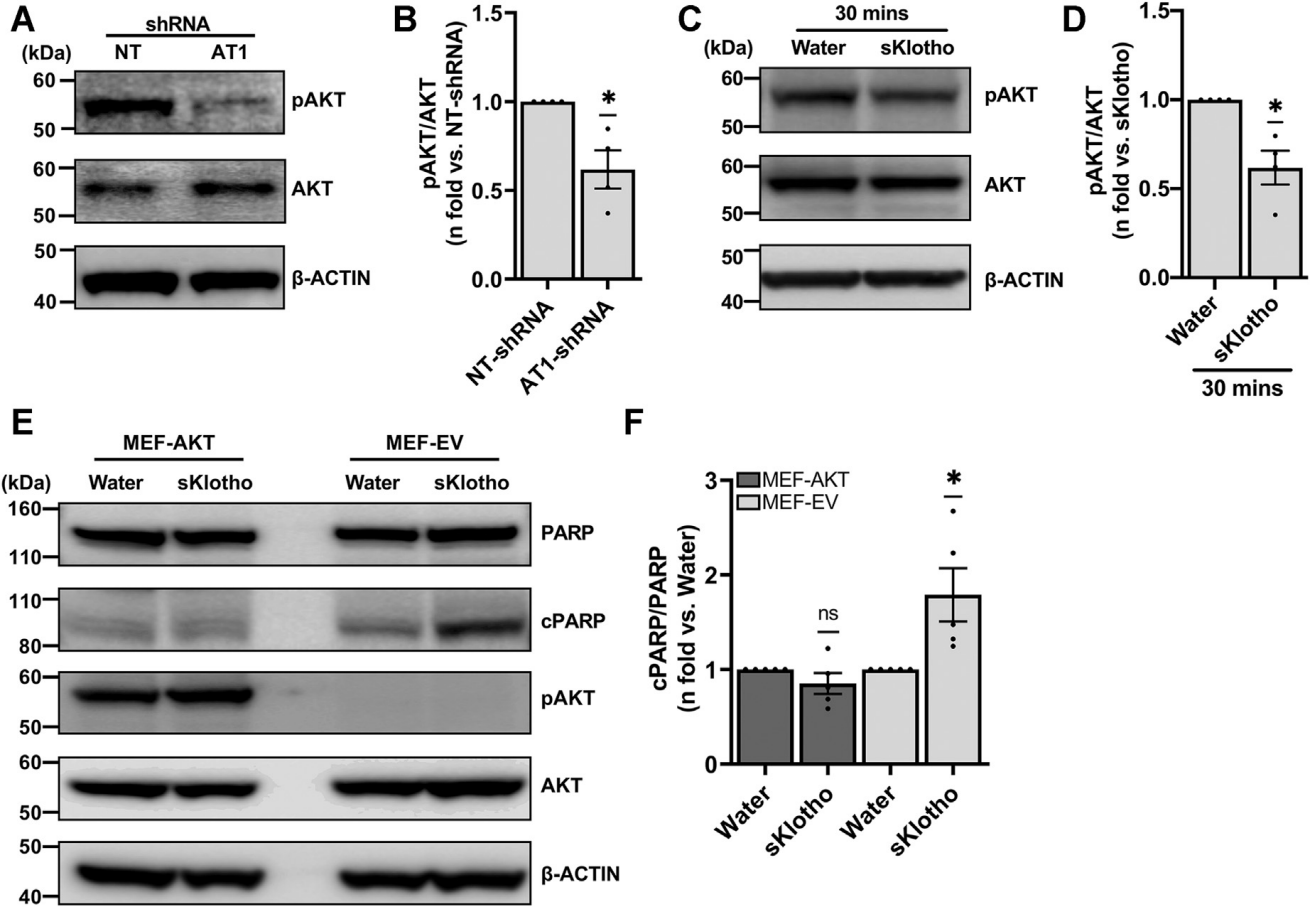
**TSC2-deficient cell death is induced by a Klotho-mediated effect on pAKT**. *A*, equal amounts of lysates extracted from NT-shRNA and AT1-shRNA ELT3V cells were analyzed by Western blot. Representative blots for pAKT, AKT, and β-ACTIN (loading control) are shown. *B*, histograms for pAKT/AKT is presented as the fold change relative to NT-shRNA–treated ELT3V cells. *C*, TSC2-deficient ELT3V cells were starved overnight and treated with vehicle control (water) or sKlotho (100 ng/ml) for indicated amount of time in serum-starved condition. Equal amounts of lysates extracted from treated cells were subjected to Western blot analysis. Representative blots for phosphorylated(p)AKT, AKT, and β-ACTIN (*loading control*) are shown. *D*, histogram for pAKT/AKT is presented as fold change relative to control (0 ng/ml sKlotho). *E*, TSC2-deficient MEF-AKT and MEF-EV cells were starved overnight and treated with vehicle control (water) or sklotho (100 ng/ml) for 24 h. Equal amounts of lysates extracted from MEF-EV– and MEF-AKT–treated cells were subjected to Western blot analysis. Representative blots for cPARP, total PARP, and β-ACTIN (loading control) are shown. (*F*) histogram for cPARP/PARP expressions in MEF-EV and MEF-AKT is presented as fold change relative to water control (0 ng/ml sKlotho). All graphs represent mean ± SEM of at least three independent experiments. Each biological replicate value is presented as a full circle. Statistical significance of ns *p* > 0.05 or ∗*p* < 0.05 were determined by one-sample *t* test. cPARP, cleaved PARP; EV, empty vector; MEF, mouse embryonic fibroblasts; NT-shRNA, nontarget shRNA; PARP, poly(ADP-ribose) polymerase; TSC, tuberous sclerosis complex.

AKT signaling pathway is associated with proliferation/survival of various cancer cells, and Klotho overexpression has been shown to decrease activation of prosurvival phospho(p)-AKT (23, 34, 36, 57). First, we found that pAKT (Ser473) levels were decreased in TSC2-deficient cells with *Agtr1* silencing (Fig. 4, *A* and *B*). We then tested the effect of sKlotho treatment at different time points on pAKT at Ser473 (not shown). Reduced activation of AKT was observed in TSC2-deficient ELT3V cells (Fig. 4, *C* and *D*) with significant decrease in pAKT level (relative to AKT) within 30 min. Additionally, to test if the induction of cell death in TSC2-deficient cells was pAKT-dependent, we treated TSC2-deficient mouse embryonic fibroblasts (MEF) transfected with EV or with constitutively active (myristoylated) AKT (MEF-AKT1) cells with sKlotho for 24 h. Similar to ELT3V cells, MEF-EV cells showed increased cell death when treated with sKlotho (Fig. 4, *E* and *F*) compared to vehicle (water) control. However, constitutive activation of AKT1 in MEF-AKT1 cells prevented the Klotho-dependent cell death (Fig. 4, *E* and *F*). These data coupled with our data showing the increased cleavage of Caspase7 upon treatment with losartan, and the known downstream effect of AKT signaling on Caspase7 cleavage (58), strongly support our hypothesis that Klotho-mediated cell death is AKT-dependent.

### AGTR inhibition suppresses TSC2-deficient xenograft tumor development *in vivo*

**Figure 5:**
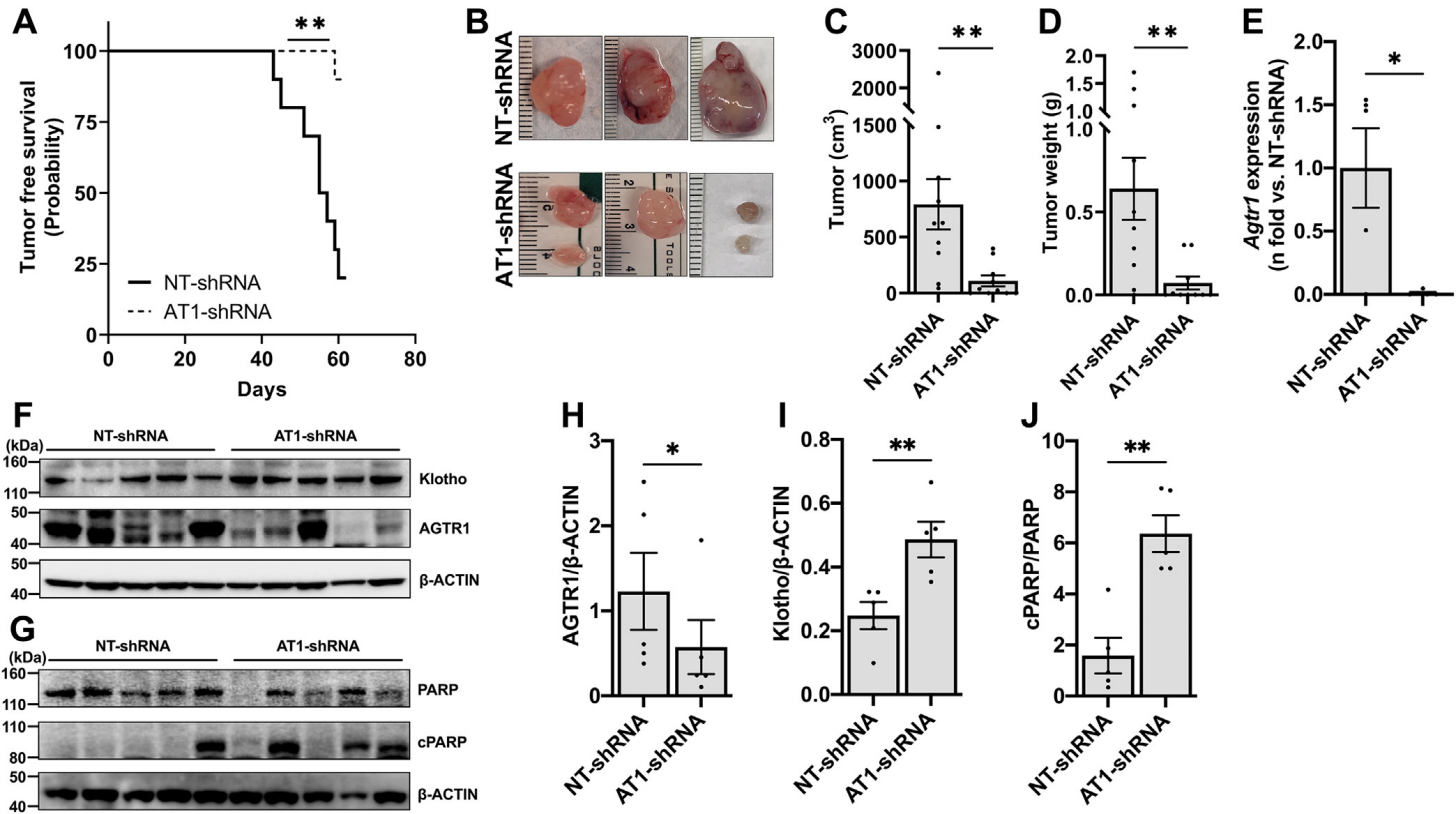
**AGTR1 blockade impairs TSC2-deficient null xenograft tumor development**. Female CB17-SCID mice were inoculated with 2.5 million control (NT-shRNA) or Agtr1 shRNA (AT1-shRNA)-transfected ELT3V cells subcutaneously into suprascapular region (*n* = 10 per group). Tumor length and width were measured daily with a caliper by a blinded investigator, and surface area was calculated. *A*, tumor-free survival analyses of mice in each group are plotted. Statistical significance ∗∗*p* < 0.01 was determined by Log-rank test. *B*, representative gross appearance of excised tumors is displayed. The scale bar represents 1 cm. Comparison of (*C*) average volume and (*D*) weight of the excised tumors. *E*, RNA was extracted from excised tumors and qPCR was performed analyzing *Agtr1* expression. *B2m* was used as housekeeping gene. *F*, representative blots for AGTR1, Klotho, and (*G*) cPARP, PARP, and β-ACTIN (loading control) for protein isolated from excised tumors are shown. Histograms for (*H*) AGTR1/β-ACTIN, (*I*) Klotho/β-ACTIN, and (*J*) cPARP/PARP expressions are presented as mean ± SEM. Each biological replicate value is presented as a full circle. Statistical significance of ∗*p* < 0.05 or ∗∗*p* < 0.01 was determined by a two-tailed *t* test. AGTR, angiotensin II receptor; cPARP, cleaved PARP; NT-shRNA, nontarget shRNA; PARP, poly(ADP-ribose) polymerase; TSC, tuberous sclerosis complex.

To evaluate the effects of AGTR1 inhibition on the growth of TSC2-deficient xenografts, TSC2-deficient NT-shRNA and AT1-shRNA cells were subcutaneously injected in immunodeficient mice. After 60 days of follow-up and monitoring, mice harboring *Agtr1* KD cells (AT1-shRNA) showed reduced tumor development compared with mice harboring NT-shRNA cells (Fig. 5*A*). Moreover, when animals were sacrificed, there was a distinct difference in gross tumor appearance (Fig. 5*B*), as well as significantly decreased tumor size (Fig. 5*C*) and weight (Fig. 5*D*) in AT1-shRNA group compared to NT-shRNA group. As expected, qPCR and Western blot analysis of lysates from tumor homogenates showed a decreased expression of AGTR1 (Fig. 5, *E*, *F*, and *H*), increased expression of Klotho (Fig. 5, *F* and *I*), and increased cPARP levels (Fig. 5, *G* and *J*). These data demonstrate that TSC2-deficient xenografts are sensitive to AGTR1 inhibition and provide rationale for future clinical trials.

**Figure 6:**
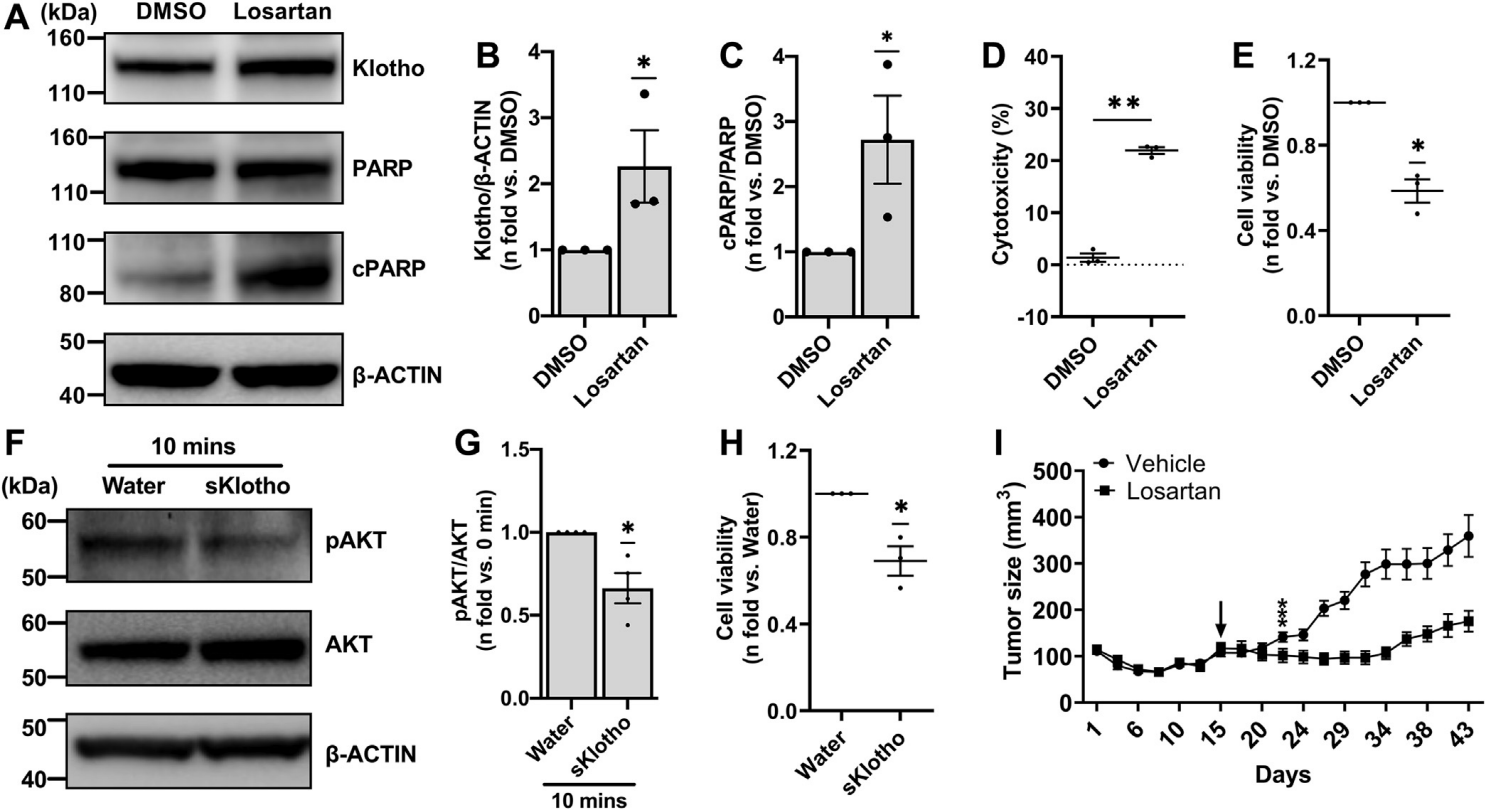
**AGTR1 blockade induces cell death in TSC2-deficient 105K cells *in vitro* and impairs xenograft tumor development *in vivo***. *A*, TSC2-deficient 105K cells were starved overnight and treated with DMSO or losartan (100 nM) for 24 h in 0.5% serum supplemented media. Equal amounts of lysates from treated cells were analyzed by Western blot. Representative blots for Klotho, cleaved PARP, total PARP, and β-ACTIN are shown. Histograms for (*B*) Klotho/β-ACTIN and (*C*) cPARP/PARP are presented as fold change relative to DMSO. *D*, scatter plot of 105K cells LDH release treated with DMSO or losartan presented as percent cytotoxicity. No drug (or water control) samples were used as “low control” for LDH measurement. *E*, cell viability was measured by the deep blue cell viability assay and values for losartan treatment are presented as percent of DMSO-treated cells. *F*, equal amounts of lysates extracted from 105K cells treated with sKlotho were subjected to Western blot analysis. Representative blots for pAKT, AKT, and β-ACTIN (loading control) are shown. *G*, histogram for pAKT/AKT is presented as fold change relative to control (0 ng/ml sKlotho). *H*, cell viability was measured using a deep blue cell viability assay and values for sKlotho treatment are presented as percent of water (vehicle)-treated cells. All graphs represent mean ± SEM of at least three independent experiments. Each biological replicate value is presented as a *full circle*. Pairwise comparisons are presented for significant differences: statistical significances of ∗*p* < 0.05 or ∗∗*p* < 0.01 were determined by (*B*, *C*, *E*, and *G–H*) one-sample t test or (*D*) two-tailed t test. *I*, growth comparison of xenograft tumors TSC2-deficient 105K tumors *in vivo* treated with vehicle (DMSO) or losartan (30 mg/kg) by oral gavage. Arrow indicates the start of losartan treatment. Statistical significance of ∗∗∗*p* <0.001 for each day was determined by two-tailed t test. AGTR, angiotensin II receptor; cPARP, cleaved PARP; DMSO, dimethyl sulfoxide; LDH, lactate hydrogenase; PARP, poly(ADP-ribose) polymerase; TSC, tuberous sclerosis complex.

Finally, we also evaluated these findings in another TSC2-deficient 105K cell line. *In vitro*, we showed that TSC2-deficient 105K cells, when treated with losartan, demonstrated increased Klotho expression (Fig. 6, *A* and *B*). We also observed increased cell death as indicated by increased cPARP levels (Fig. 6, *A* and *C*), increased LDH release (Fig. 6*D*), and decreased cell viability (Fig. 6*E*) with losartan treatment. Moreover, treatment with sKlotho resulted in decreased AKT phosphorylation (Fig. 6, *F* and *G*). Further, and like TSC2-addback ELT3T cells, inhibition of AGTR1 in TSC2-addback 105K cells with losartan treatment showed no difference in Klotho and cPARP levels (Fig. S5, *A*–*C*) as well as in percent LDH release compared to DMSO treatment (Fig. S5*D*). We also evaluated the activity of losartan in an immunodeficient mouse model bearing subcutaneous TSC2-deficient 105K xenograft tumors (7, 9). Mice harboring TSC2-deficient 105K xenograft tumors treated with vehicle control (DMSO) showed progressive tumor growth. In contrast, mice treated with losartan showed a persistent stabilization in tumor size (Fig. 6*I*).

## Discussion

In this study, we demonstrate that there is an important role for the AGTR1–Klotho axis in TSC2-deficient cells. Our data provide evidence that blocking AGTR1 using losartan, a commercially available angiotensin receptor blocker, induces cell death in TSC2-deficient cells from two different origins, ELT3 cells derived from an TSC2-deficient Eker rat uterine leiomyoma (46), and 105K cells derived from a renal tumor in a TSC2 heterozygous mouse (59). It is important to note that losartan has additional off-target receptor-independent cellular effects especially on transforming growth factor-β signaling (50, 51, 52, 53). However, shRNA-mediated knockdown of AGTR1 resulted in similar effects to losartan on TSC2-deficient cell death, indicating that effects of losartan are less likely due to an off-target effect and more likely due to inhibition of the AGTR1. Consistently, losartan treatment or shRNA-mediated receptor silencing significantly reduced tumor burden in an immunodeficient xenograft model, suggesting a potential role for targeting AGTR1 in LAM and other manifestations of TSC. Interestingly, our data show that there are no differences in AGTR1 expression in TSC2-deficient and TSC2-addback cells. However, inhibiting AGTR1 resulted in cell death only in TSC2-deficient cells but not TSC2-addback cells, suggesting that only TSC2-deficient cells require a continued AGTR1 signaling for survival. Our data pave the way to potentially use this vulnerability to target tumors, resulting from tuberin deficiency and hyperactivation of the mTORC1 pathway.

The potential therapeutic effects of targeting renin-angiotensin in LAM and TSC have been demonstrated in retrospective studies of lung function (21). Patients with TSC2-polycystic kidney disease 1 deletion syndrome and hypertension treated with ACE inhibitors or angiotensin receptor blockers had decreased renal AML development compared to control (22). *In vitro* evidence suggested that stimulation of AGTR1 by angiotensin II drives VEGF-A secretion in mTORC1-activated, TSC2-deficient angiomyolipoma cells, leading to increased cell proliferation, which was shown to be blocked by valsartan, an AGTR1 inhibitor (22). The losartan concentration that we used *in vitro* (0.0479 μg/ml) is well within the 1 μg/ml serum concentration achieved with the current therapeutic dosage of 100 mg orally daily (60). Losartan has an excellent lung bioavailability (61); however, our *in vivo* proof of concept experiments using a 30 mg/kg dose would be the equivalent to ∼2.4 mg/kg human equivalent dose (62), a higher losartan dose than the commonly used 100 mg orally daily (61). In clinical practice and based on their use for hypertension, the safety profile of angiotensin receptor blockers is well-documented. In addition, losartan has been used in clinical trials at 200 mg orally daily with no difference in adverse side effects between the high dose (200 mg daily) compared to the standard dose of 100 mg orally daily (63). Collectively, these previous data and our data provide compelling evidence of the viability of a therapeutic strategy using angiotensin receptor blockade, perhaps in early diseases affecting patients with LAM or TSC.

Our data also indicates that the cytocidal effects triggered by blocking the AGTR1 are mediated by an increase in Klotho protein expression. Klotho is a tumor suppressor that has previously been shown to interact with the mTOR pathway (40). Klotho has previously been implicated in lung disease with effects on muco-ciliary clearance (64), airway inflammation (65), recovery from acute lung injury (66) and interstitial lung disease (67, 68) amongst others. Our data show the importance of Klotho upregulation in TSC2-deficient cells. *In vitro*, both membrane and sKlotho induces TSC2-deficient cell death. We also showed that Klotho silencing rescues the cell death phenotype induced by losartan treatment, suggesting Klotho is necessary and sufficient for regulating TSC2-deficient cell survival. Additionally, blockade of the AGTR1 also results in an increase in Klotho mRNA levels and the effects are specific to TSC2-deficient cells. Both membrane and sKlotho influence several signaling pathways, including insulin-like growth factor 1–mediated PI3K/AKT signaling pathway; notably, an increase in Klotho has been shown to decrease phosphorylation of AKT, a known prosurvival pathway (34, 36, 69, 70). The role of AKT in TSC2-deficient cells remains understudied. In TSC2-deficient cells, mTORC1 hyperactivation negatively impacts AKT phosphorylation *via* inhibition of the mTORC2 complex and disruption of PI3K signaling. However, minimal activation of mTORC2 is sufficient to phosphorylate AKT, and AKT rebound phosphorylation when these cells are treated with rapamycin represents a key prosurvival pathway in TSC2-deficient cells (57). While much remains to be discovered regarding the regulation of AKT signaling by the TSC1/2 complex (71), our data suggest a prosurvival role for AKT in TSC2-deficient cells, as a decrease in pAKT induced by Klotho treatment was associated with increased cell death. Consistent with this result, Klotho-mediated suppression of AKT phosphorylation was associated with increased cell death, and expression of constitutively phosphorylated AKT protected TSC2-deficient cells from Klotho-mediated cell death. Taken together, these data suggest that AGTR1 inhibition drives Klotho expression, which decreases phosphorylation of AKT, a known prosurvival pathway, resulting in TSC2-deficient cell death.

In conclusion, consistent with previous data showing the importance of AGTR1 (20, 21) in LAM and AML (22), this study demonstrates both *in vitro* and *in vivo* and offers the scientific rationale to target AGTR1 in LAM and in TSC. We delineated the importance of AGTR1 signaling in TSC2-deficient cells and demonstrated that AGTR1 blockade could be a potential therapy in LAM and TSC, especially early in the disease when there could be a potential hesitation to commit patients to lifelong therapy with inhibitors of the mTORC1 pathway. Because AGTR1 inhibitors have a remarkable safety profile, evaluating their efficacy in clinical trials in LAM, AML, and other manifestations of TSC is warranted.

## Experimental procedures

### Cell lines, cell culture, and reagents

Cells were cultured in Dulbecco’s modified Eagle medium (DMEM) supplemented with 10% fetal bovine serum at 37 °C in a humidified 5% CO_2_ atmosphere. TSC2-deficient ELT3V and TSC2-addback ELT3T cells are derived from Eker rat uterine leiomyoma, as described previously (46). TSC2-deficient and TSC2-addback cystadenoma 105K cell lines were obtained from Dr Elizabeth Henske’s laboratory (59). TSC2-deficient MEF infected with pBabe-Puro-Myr-Flag-AKT1, to generate TSC2-deficient MEF with myristoylated AKT1 (MEF-AKT1), and control TSC2-deficient MEF (MEF-EV) infected with EV were obtained from Dr Carmen Priolo’s laboratory (72). Myristoylation directs AKT to the membrane, keeping it constitutively phosphorylated at both Ser473 and Thr308 sites (72, 73). Cells were serum starved overnight before treatment with indicated drugs/compounds, including losartan (100 nM (47, 74); Tocris Bioscience), DMSO vehicle (Sigma-Aldrich), water (vehicle), or Klotho (100 ng/ml (55, 56); R&D Systems) for indicated durations.

### Lentiviral expression system

To establish stable *Agtr1* KD TSC2-deficient ELT3V cells, we obtained a psi-LVRU6GH vector harboring nontargeting shRNA oligonucleotide sequence (NT-shRNA) and a psi-LVRU6GH vector harboring Agtr1-targeting shRNA oligonucleotides sequences (AT1-shRNA or AT1-shRNA-2). The shRNA oligonucleotide sequences are listed in Table S1. Each vector consisted of EGFP and hygromycin selection markers. Lentiviral particles were produced by cotransfecting each shRNA vector together with third generation lentiviral packaging plasmids (Addgene), including pMDLg/pRRE (Addgene plasmid #12251), pRSV-Rev (Addgene plasmid #12253), and pCMV-VSV-G (Addgene plasmid #8454) into 293T cells using Lipofectamine 3000 reagent (Thermo Fisher Scientific). Lentiviral packaging plasmids were gifts from Didier Trono (75). *Agtr1* shRNA oligonucleotide constructs cloned into psi-LVRU6GH vectors were designed and generated by GeneCopoeia, Inc. The media were replaced 16 h posttransfection and supernatants were collected 48 h posttransfection, which were used to infect cultured cells for 48 h. The culture media was replaced with 100 μg/ml hygromycin B (Sigma-Aldrich) containing media every 2 days for 10 days. Complete cell death of no virus control was ensured before the end of the selection. After selection, knockdown efficiency was assessed using qPCR and Western blot analyses of endogenous *Agtr1* mRNA and AGTR1 protein expression.

### RNA interference

Predesigned MISSION siRNA for *Klotho* gene (Klotho siRNA) and MISSION siRNA Universal Negative control (Scr siRNA) were purchased from Sigma-Aldrich. RNA interference was performed in ELT3V cells with the indicated concentration of each siRNA using Lipofectamine RNAiMAX reagent (Thermo Fisher Scientific) and Opti-MEM (Thermo Fisher Scientific). Media containing transfection mix was replaced with no serum media 8 h posttransfection. Cells were starved overnight and treated with DMSO or losartan in 0.5% serum media for additional 24 h. Total siRNA transfection duration before harvest was 48 h. Sequences and concentrations for siRNA used are listed in Table S1.

### Plasmid

For transient expression of Klotho (membrane), sequence cloned into pcDNA3.1/V5/His-TOPO expression vector was a gift from Dietz (Addgene plasmid # 17712; http://n2t.net/addgene:17712; RRID:Addgene_17712) (73). EV, pcDNA3.1 was used as control. Two micrograms of each plasmid was transfected into ELT3V cells using Lipofectamine 3000 reagent (Thermo Fisher Scientific) and Opti-MEM (Thermo Fisher Scientific). Transfection media was replaced at 8 h posttransfection and cells were harvested for analysis at 48 h after transfection.

### Quantitative RT-PCR

Total RNA from cultured cells for each experiment were isolated using RNeasy Plus Mini kit (Qiagen), and cDNA was synthesized using amfiRivert cDNA Synthesis Platinum Master Mix (GenDEPOT), according to the manufacturers’ protocol. Real-time qPCR was performed using iTaq Universal SYBR Green Supermix (Bio-Rad Laboratories). Primers used for RT-qPCR for target genes and housekeeping genes are listed in Table S2.

### Protein extraction and Western blotting

Cells in culture dishes were placed on ice, washed twice with cold PBS, scraped, and centrifuged into pellets. Then, the cell pellets were lysed in RIPA lysis buffer (Invitrogen) supplemented with protease and phosphatase inhibitors (Invitrogen) for 30 min with constant agitation and centrifuged at 13,000*g* for 15 min at 4 °C. The supernatants were collected for Western blot analysis. Equal amounts of total protein were loaded onto NuPage 4% to 12% Bis-Tris Protein Gels (Invitrogen) and then subsequently immunoblotted with the primary antibodies listed in Table S3.

### LDH colorimetric and deep blue cell viability assays

To investigate if treatment with losartan or sKlotho induced cytotoxicity, LDH-Cytox Assay Kit (BioLegend) was used following the “homogeneous assay using viable cells” protocol for LDH measurement. Additionally, the Deep Blue Cell Viability kit (BioLegend) was used as a secondary measure of cytotoxicity induced by losartan or sKlotho treatment. For both assays, cells were cultured in a 96-well plate for 24 h, serum starved overnight, and treated with control (Water), DMSO, losartan, or sKlotho in 0.5% serum media for 24 h. For shRNA-transduced cells, an equal number of NT-shRNA or AT1-shRNA or AT1-shRNA-2 cells were cultured in a 96-well plate for 24 h, serum starved overnight, and media replaced with 0.5% serum media for additional 24 h. The measurements (luminescence or fluorescence readings) were taken and analyzed according to the manufacturers’ protocols. For LDH assay, water-treated samples were used as “low control” for percent cytotoxicity calculation. Comparisons were made between DMSO and losartan or water and sKlotho. For shRNA-transduced cells LDH assay, NT-shRNA samples were used as “low control” for percent cytotoxicity calculation. For viability assay, fluorescence levels for losartan, sKlotho, or AT1-shRNA/AT1-shRNA-2 were determined relative to DMSO, water, or NT-shRNA respectively.

### Animal studies

All animal procedures were performed according to protocols approved by the Institutional Animal Care and Use Committee at Brigham and Women’s Hospital. LAM is a disease that affects women; therefore, all animal studies were performed on female mice. For the *in vivo* xenograft study, female immunodeficient C.B17 SCID mice (6–7 weeks old) were obtained from Taconic Biosciences and randomly divided into two groups for injection (NT-shRNA or AT1-shRNA). A total of 2.5 × 10^6^ NT-shRNA or AT1-shRNA cells in 200 μl cell culture media supplemented with 25% Matrigel (Corning Inc) were subcutaneously injected into the suprascapular area (10 mice/group). Mice were then closely monitored for body weight and general health status every 3 days until the tumor development and every other day after. Tumors’ sizes/volumes were measured using calipers and calculated using the standard equation (1/2)(L × W^2^), where W is the smaller side of the tumor. All mice were euthanized when one of the mice met the institutional euthanasia criteria for xenograft tumor size (*i.e.*, ≥2 cm in diameter). The tumors were removed, photographed, weighed, and cut into sections for qPCR and Western blot. Total RNA was isolated using a RNeasy Plus Mini kit (Qiagen), according to the manufacturer’s protocol and total protein was isolated using RIPA lysis buffer supplemented with protease and phosphatase inhibitors as previously described in section “Protein extraction and Western blotting”.

For *in vivo* losartan treatment, the experiment was conducted in collaboration with the TSC Alliance Preclinical Consortium. A total of 2.5 × 10^6^ *TSC2-deficient* cystadenoma 105K cells in a 1:1 ratio of DMEM and Matrigel were injected into female nude mice *via* subcutaneous injection. Fourteen mice were used per treatment group. Once the average xenograft tumor volume reached about 100 mm^3^ (day 15), mice were administered with losartan (30 mg/kg) or vehicle control daily by p.o. route for 28 consecutive days. After the treatment phase, 10 mice selected at random were monitored for an additional 28-day period for tumor regrowth analysis.

### Statistical analyses

All data are presented as mean ± SEM of at least three independent experiments or biological replicates. Details of statistical tests and significance for each experiment are presented in the corresponding figure legends. One-sample *t* test was used to compare control and treatment groups when data were normalized to the control group (*i.e.*, mean of control group expressed as 1). *t* test (two-tailed) was utilized to compare means between any two groups. For *in vivo* data, *t* test (two-tailed) was used to compare data between two groups. Log-rank (Mantel-Cox) test was utilized to compare the tumor-free survival. All analyses were done using GraphPad Prism 9.2.0 (GraphPad Prism Software, San Diego, CA, www.graphpad.com). *p*-value of less than 0.05 was considered significant.

## Data availability

All the relevant data are contained within the article and the supporting information.

## Supporting information

This article contains supporting information.

## Conflict of interest

The authors declare that they have no conflicts of interest with the contents of this article.
