# Supplementary material for: Angiotensin II receptor type 1 blockade regulates Klotho expression to induce TSC2-deficient cell death
Source: J Biol Chem. 2022 Oct 8;298(11):102580. doi: 10.1016/j.jbc.2022.102580 (PMC9661712; doi:10.1016/j.jbc.2022.102580)
Supplement: Supplementary Material [file mmc1.doc]

**Angiotensin II receptor type 1 blockade regulates Klotho expression to induce TSC2-deficient cell death**

Shikshya Shrestha^1^, Elio Adib^1^, Jewel Imani^1^, Dean J. Aguiar^2^, Anthony M. Lamattina^1^, Dereje D. Tassew^1^, Elizabeth P. Henske^1^, Mark A. Perrella^1^, Carmen Priolo^1^, Souheil El-Chemaly^1^

1. Division of Pulmonary and Critical Care Medicine, Brigham and Women’s Hospital, Harvard Medical School, Boston, MA
2. TSC Alliance, Silver Spring, MD

**Correspondence**

Souheil El-Chemaly, MD, MPH

Brigham and Women’s Hospital

75 Francis Street, Boston, MA 02115

617-732-6869

[Sel-chemaly@bwh.harvard.edu](mailto:Sel-chemaly@bwh.harvard.edu)

Materials included:

Supplementary Figure 1

Supplementary Figure 2

Supplementary Figure 3

Supplementary Figure 4

Supplementary Figure 5

Supplementary Table S1

Supplementary Table S2

Supplementary Table S3


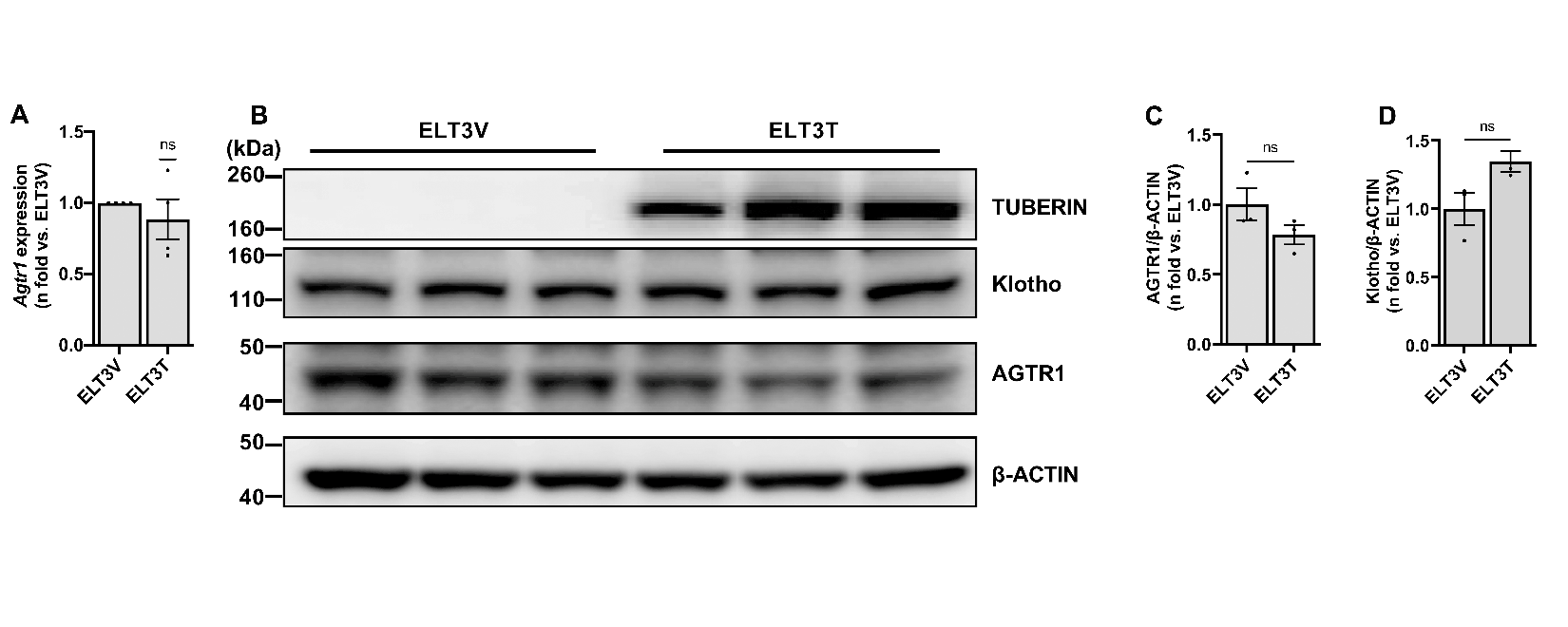


**Supplementary Figure 1: AGTR1 expression in TSC2-deficient and *Tsc2*-addback cells.**

**(A)** Histogram for qPCR analysis of *Agtr1* expression in TSC2-deficient ELT3V cells compared to TSC2-addback ELT3T is presented. *B2m* was used as a housekeeping gene. **(B)** Equal amounts of protein from whole cell lysates extracted from ELT3V and ELT3T cells were subjected to western blot analysis. Representative blots for TUBERIN, AGTR1, Klotho and β-ACTIN (loading control) are shown. Histograms for **(C)** AGTR1/β-ACTIN and **(D)** Klotho/β-ACTIN are expressed as the fold change relative to ELT3V cells. All graphs represent mean±SEM of at least three independent experiments. Each biological replicate value is presented as a full circle. Statistical significance of ns P > 0.05 for each histogram was determined by one sample t-test.


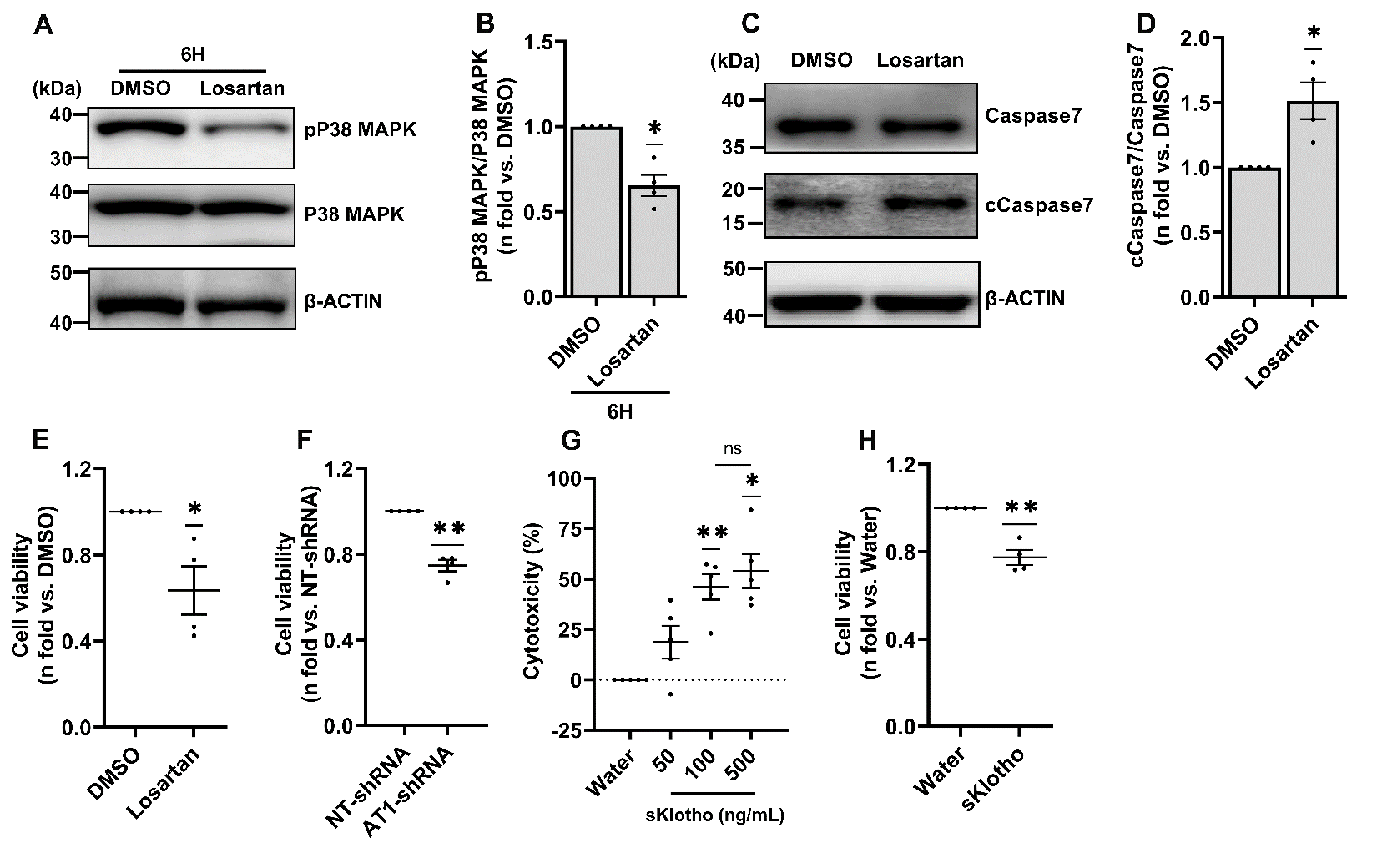


**Supplementary Figure 2: Effects of AGTR1 inhibition TSC2-deficient cell survival.** **(A)** Representative blots for P38 MAPK, phospho(p)-P38 MAPK and β-ACTIN (loading control) and **(B)** histogram for p-P38 MAPK/P38 MAPK (expressed relative to DMSO) in ELT3V cells treated with losartan (100 nM) or DMSO control for 6 hours are shown. **(C)** Representative blots for cleaved(c)-Caspase7, Caspase7 and β-ACTIN (loading control) and **(D)** histogram for cCaspase7/Caspase7 (expressed relative to DMSO) in ELT3V cells treated with losartan (100 nM) or DMSO control for 24 hours are shown. Cell viability was measured by deep blue cell viability assay **(E)** in ELT3V cells treated with DMSO or losartan (100 nM) for 24 hours and **(F)** in ELT3V cells targeted with control (NT-shRNA) or AT1-shRNA. (**G)** LDH release assay was performed after incubation of ELT3V cells with different concentrations of sKlotho. **(H)** Cell viability was measured by deep blue cell viability assay in ELT3V cells treated with water or sKlotho (100 ng/ml) for 24 hours. All graphs represent mean±SEM of at least three independent experiments. Each biological replicate value is presented as a full circle. Statistical significance of * P < 0.05 or ** P < 0.01 was determined by one sample t-test.


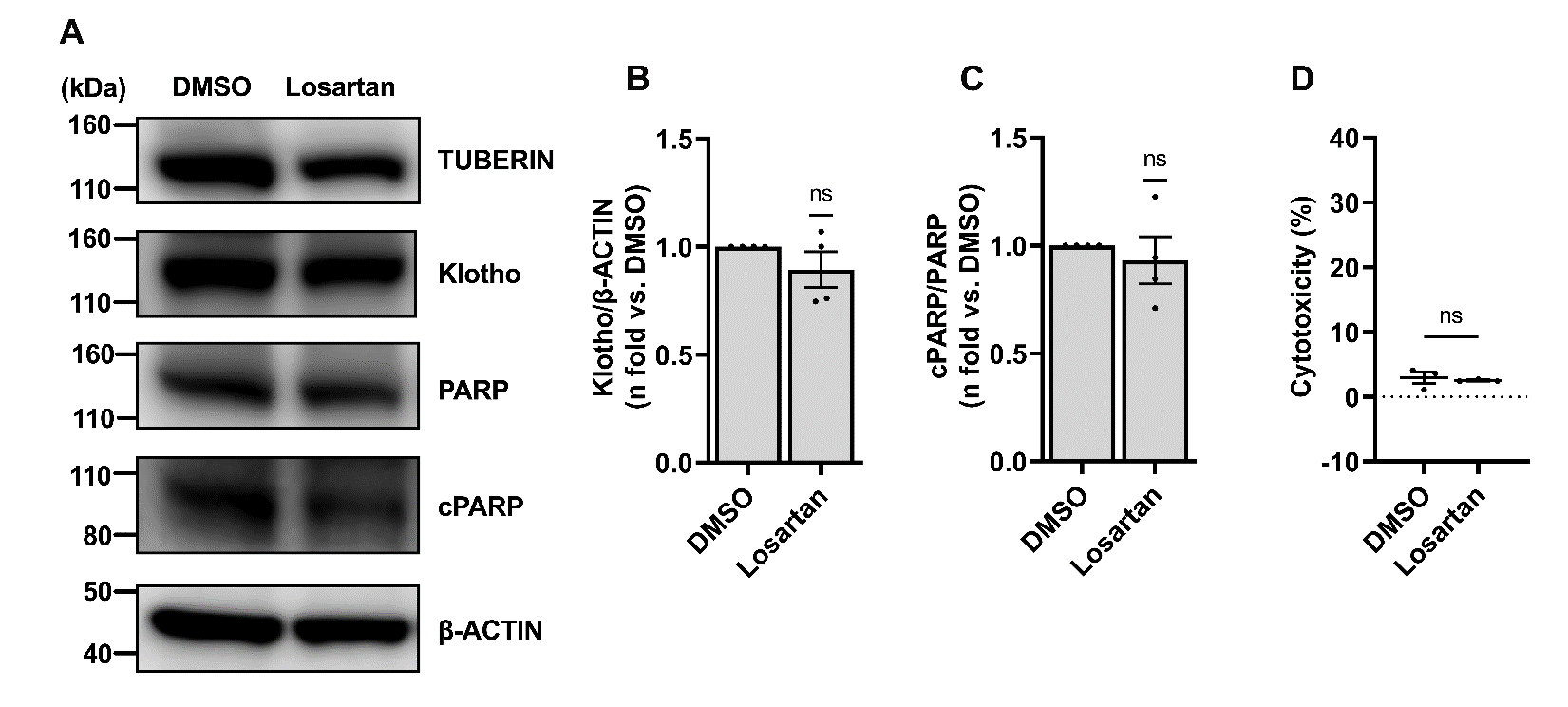


**Supplementary Figure 3: Inhibition of AGTR1 does not induce Klotho–dependent cell death in TSC2-addback ELT3T cells *in vitro*.**

**(A)** TSC2-addback ELT3T cells were serum starved overnight and treated with DMSO or losartan (100 nM) for 24 hours in 0.5% serum supplemented media. Equal amounts of protein lysates from treated cells were subjected to western blot analysis. Representative blots for TUBERIN, Klotho, PARP, cPARP and β-ACTIN (loading control) for ELT3T cells are presented. Histograms for **(B)** Klotho/β-ACTIN and **(C)** cPARP/PARP are presented as fold change relative to DMSO. **(D)** Graph of LDH release presented as percent cytotoxicity after exposure of ELT3T to DMSO or losartan treatment. All graphs represent mean±SEM of at least three independent experiments. Each biological replicate value is presented as a full circle. Statistical significance of ns P > 0.05 was determined by **(B-C)** one sample t-test or **(D)** two-tailed t-test.


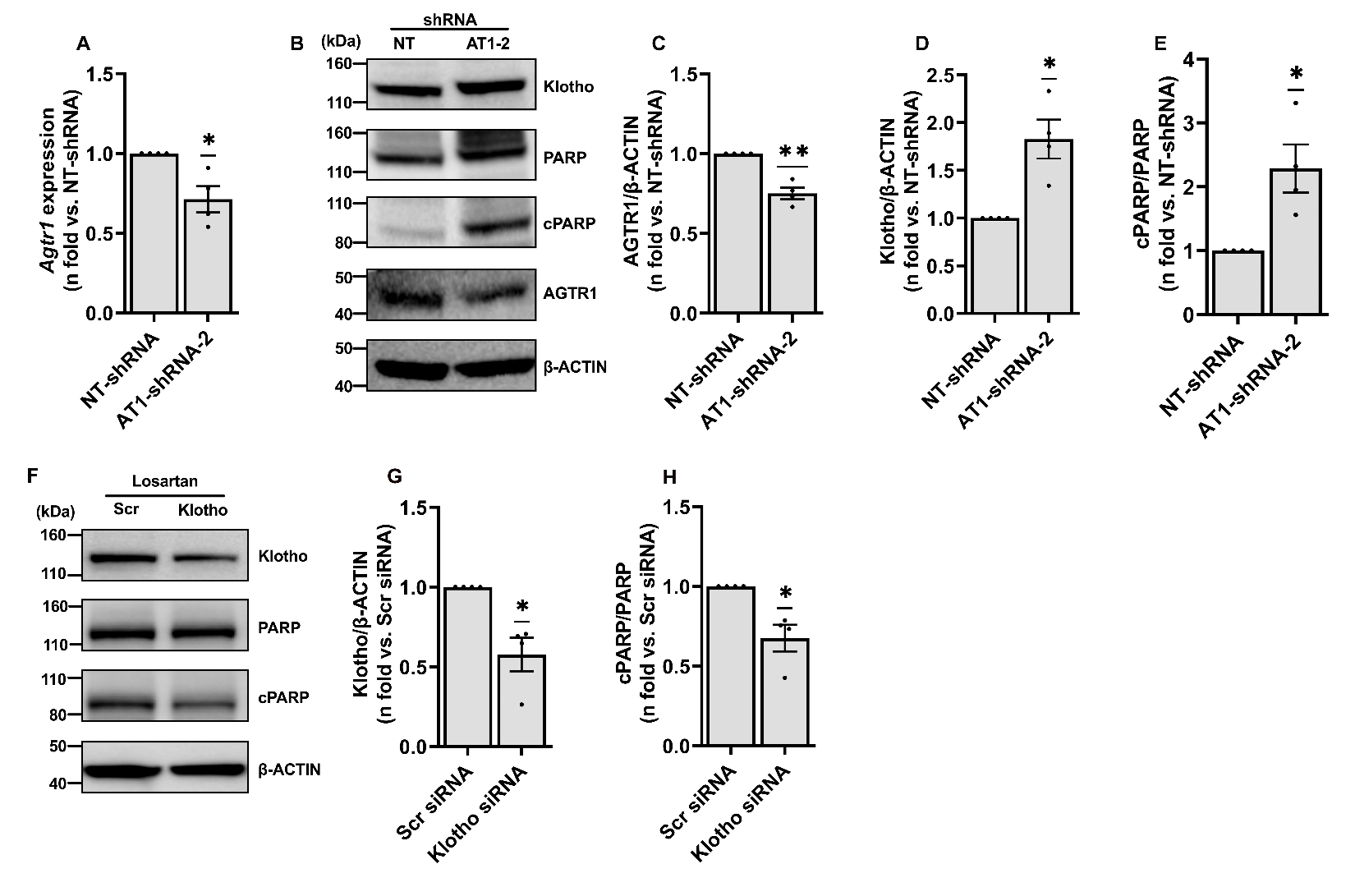


**Supplementary Figure 4: TSC2-deficient cell death induced by AGTR1 inhibition and rescue by Klotho silencing validated by a second shRNA and siRNA. (A)** Histogram for qPCR analysis of *Agtr1* expression in ELT3V cells targeted with control (NT-shRNA) and AT1-shRNA-2 are presented. *B2m* was used as a housekeeping gene. **(B)** Equal amounts of whole-cell lysates of NT-shRNA and AT1-shRNA-2 cells were analyzed by western blot. Representative blots for AGTR1, Klotho, cPARP, PARP and β-ACTIN (loading control) are shown. Histograms for **(C)** AGTR1/β-ACTIN **(D)** Klotho/β-ACTIN and **(E)** cPARP/PARP are expressed as the fold change relative to NT-shRNA cells. All graphs represent mean±SEM of at least three independent experiments. **(F)** Representative blots for Klotho, PARP, cPARP and β-ACTIN (loading control) for ELT3V cells transfected with second Klotho siRNA and treated with losartan for 24 hours. Histograms for **(G)** Klotho/β-ACTIN and **(H)** cPARP/PARP are expressed as the fold change relative to Scr siRNA transfected cells. Each biological replicate value is presented as a full circle. Statistical significance of * P < 0.05 or ** P < 0.01 was determined by one sample t-test.


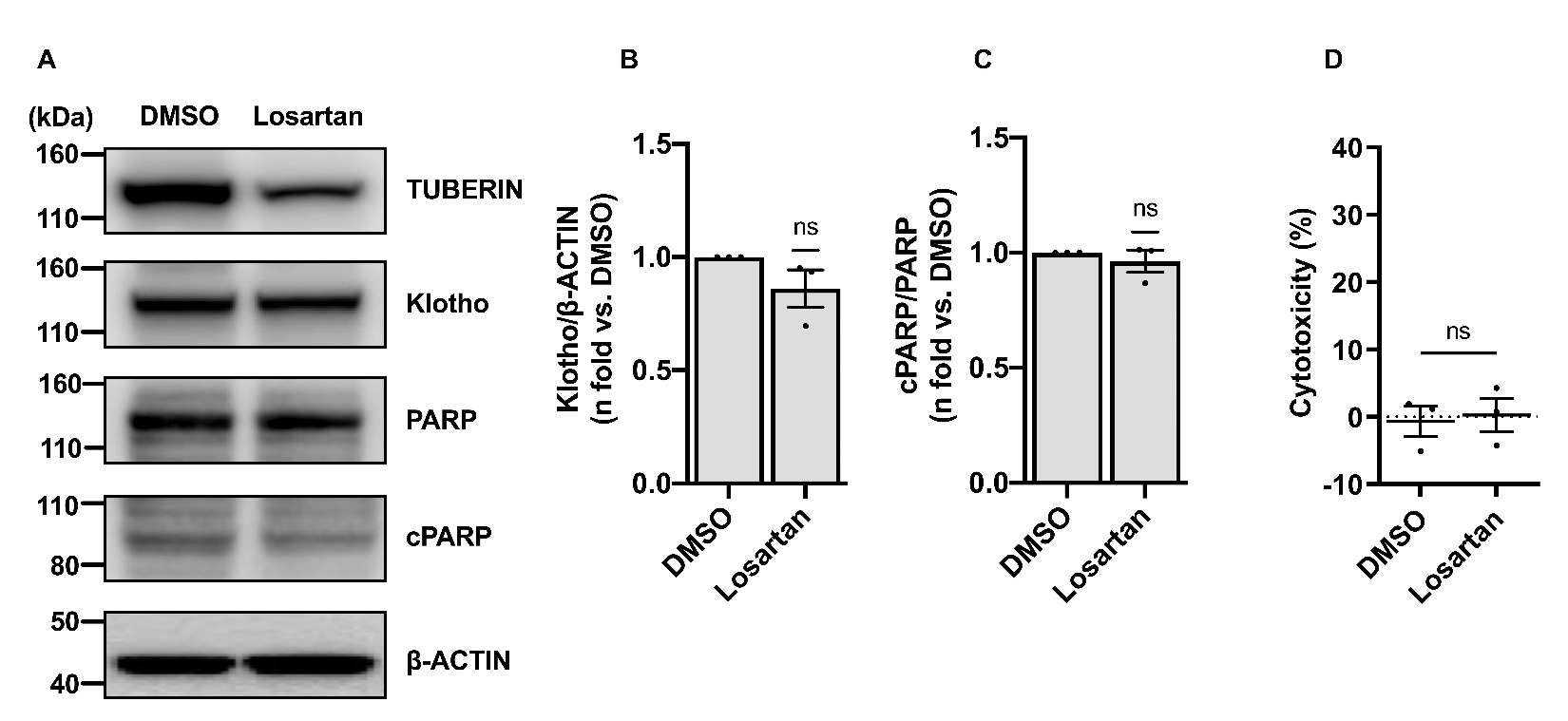


**Supplementary Figure 5: Inhibition of AGTR1 does not induce Klotho–dependent cell death in TSC2-addback 105K cells *in vitro*.**

**(A)** TSC2-addback 105K cells were serum starved overnight and treated with DMSO or losartan (100 nM) for 24 hours in 0.5% serum supplemented media. Equal amounts of protein lysates from treated cells were subjected to western blot analysis. Representative blots of TUBERIN, Klotho, PARP, cPARP and β-ACTIN (loading control) for 105K cells are shown. Histograms for **(B)** Klotho/β-ACTIN and **(C)** cPARP/PARP are presented as fold change relative to DMSO. **(D)** Graph of LDH release presented as percent cytotoxicity treatment after exposure of TSC2-addback 105K cells to DMSO or losartan treatment. All graphs represent mean±SEM of at least three independent experiments. Each biological replicate value is presented as a full circle. Statistical significance of ns P > 0.05 for each graph was determined by one sample t-test.

**Supplementary Table S1. siRNA and shRNA oligonucleotide sequences used**

| **Target gene** | **Oligo** | **Sequence (5’-3’)** |
| --- | --- | --- |
| Klotho | siRNA | guucauaauggaaagcuua[dT][dT] |
| Klotho (2) | siRNA | gucagaagucucuugacuu[dT][dT] |
| Agtr1 sh (AT1-shRNA) | shRNA | gctctaaagaaggcttatgaa |
| Agtr1 sh-2 (AT1-shRNA-2) | shRNA | ccaccgaaatgtatacttcat |
| Non-targeting sh-NT | shRNA | gcttcgcgccgtagtctta |

**Supplementary Table S2. Primers used for RT-qPCR**

| **Gene** | **NCBI Accession#** | **Species** | | **Sense 5’-3’** | **Antisense 5’-3’** |
| --- | --- | --- | --- | --- | --- |
| rAgtr1a | NM_030985.4 | *Rattus norvegicus* | | tcaccaggtcaagtggatttcg | gtcagccaaggcgagattga |
| rKlotho | NM_031336.2 | *Rattus norvegicus* | | caactacattcaagtggacc | cagtaaggttttctcttcttgg |
| rB2m | NM_012512 | *Rattus norvegicus* | | actggtctttctacatcctg | agatgattcagagctccatag |
| mAgtr1a | NM_177322.3 | *Mus musculus* | | aatatggaagtgcctcgctg | ctttcatatgttaagtccgggaga |
| mKlotho | NM_013823.2 | *Mus musculus* | | atggcgactacccagagagt | agagagtagtgtccacttgaacg |
| mB2m | NM_009735.3 | *Mus musculus* | | gtatgctatccagaaaaccc | ctgaaggacatatctgacatc |
|  | | |  | | |

**Supplementary Table S3**. **List of antibodies used for western blots**

| **Target** | **Company** | **Catalog#** | **MW** |
| --- | --- | --- | --- |
| Agtr1 | Origene | TA328665 | 41 |
| pAKT | Cell Signaling | #9271 | 60 |
| AKT | Cell Signaling | #4691 | 60 |
| β-Actin AC-74 | Sigma Aldrich | A2228 | 42 |
| cCaspase7 | Cell Signaling | #9491 | 20 |
| Caspase7 | Cell Signaling | #9492 | 35 |
| Klotho | Abcam | ab203576 | 116 |
| P38 MAPK | Cell Signaling | #8690 | 38 |
| pP38 MAPK | Cell Signaling | #9211 | 38 |
| cPARP | Cell Signaling | #9545 | 89 |
| PARP | Cell Signaling | #9542 | 116 |
| Tuberin/TSC2 D93F12 | Cell Signaling | #4308 | 200 |
